# Tertiary Amine Oxide-Containing Zwitterionic Polymers: From Material Design to Biomedical Applications

**DOI:** 10.3390/pharmaceutics17070846

**Published:** 2025-06-27

**Authors:** Jian Shen, Tao Sun, Yunke Bi

**Affiliations:** 1 College of Chemistry, Chemical and Environmental Engineering, Weifang University, Weifang 261061, China; shenjian@wfu.edu.cn; 2 Key Laboratory of Smart Drug Delivery/Innovative Center for New Drug Development of Immune Inflammatory Diseases (Ministry of Education), Minhang Hospital, State Key Laboratory of Medical Neurobiology and MOE Frontiers Center for Brain Science, Department of Pharmaceutics, School of Pharmacy, Fudan University, Shanghai 201203, China; 3 National Key Laboratory of Advanced Drug Formulations for Overcoming Delivery Barriers, Fudan University, Shanghai 201203, China; 4 Quzhou Fudan Institute, Quzhou 324003, China; 5 Department of Neurosurgery, Shanghai General Hospital, School of Medicine, Shanghai Jiao Tong University, Shanghai 200025, China

**Keywords:** tertiary amine oxide, zwitterionic polymer, drug delivery, antifouling properties, dynamic cell membrane affinity, active transcytosis

## Abstract

Tertiary amine oxide (TAO)-containing zwitterionic polymers are a class of zwitterionic materials formed by the oxidation of tertiary amine groups. In recent years, polymers such as poly(2-(N-oxide-N,N-diethylamino)ethyl methacrylate) (OPDEA) have gained significant attention due to their unique antifouling properties, dynamic cell membrane affinity, and responsiveness to microenvironments. These characteristics have made them promising candidates in drug delivery, antibiofouling, and precision therapy. Compared to traditional polyethylene glycol (PEG), these polymers not only exhibit long-circulation properties but can also overcome biological barriers through active transport mechanisms, making them a research hotspot in the field of next-generation biomaterials. This review comprehensively summarizes the recent advancements in this field, covering aspects such as the synthesis, properties, applications, and mechanisms of TAO-containing zwitterionic polymers.

## 1. Introduction

**Scheme 1:**
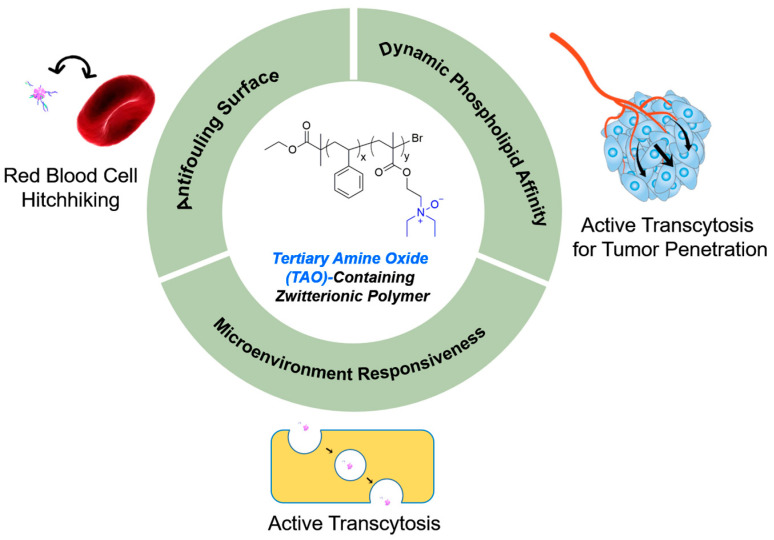
Properties of TAO-containing zwitterionic polymers, including their molecular structure features, zwitterionic characteristics, dynamic cell membrane affinity, and potential uses in drug delivery and other biomedical fields.

In the rapidly evolving landscape of biomedical materials [1], the quest for advanced polymers that can transcend the limitations of conventional nanomedicines has become increasingly urgent [2]. While significant progress has been made in drug delivery systems, the field still grapples with challenges such as poor tumor penetration, nonspecific protein adsorption, and the failure to overcome complex biological barriers [3]. Against this backdrop, tertiary amine oxide (TAO)-containing zwitterionic polymers have emerged as a novel class of materials that hold the potential to redefine the frontiers of nanomedicine [4]. As illustrated in Scheme 1, these polymers possess unique properties such as zwitterionic characteristics and dynamic cell membrane affinity [5], indicating that they have promise in drug delivery and other biomedical fields. Scheme 1 highlights the key aspects of TAO-containing zwitterionic polymers, including their molecular structure features and potential application scenarios. The phospholipid affinity segment in Scheme 1 refers to the ability of these polymers to weakly bind to cell membrane phospholipids, while the transcytosis part indicates their capability to trigger active cellular uptake mechanisms. These properties collectively contribute to the enhanced performance of TAO-containing zwitterionic polymers in biomedical applications.

These polymers, particularly poly(2-(N-oxide-N,N-diethylamino)ethyl methacrylate) (OPDEA) [6], have garnered substantial attention due to their unique physicochemical properties. The synthesis of OPDEA involves a meticulous two-step process. Initially, a precursor polymer containing tertiary amine groups is synthesized via controlled polymerization techniques such as Atom Transfer Radical Polymerization (ATRP). Subsequently, the tertiary amine groups undergo oxidation to introduce N-oxide groups. This synthesis strategy not only ensures precise control over the molecular structure but also lays the foundation for further functionalization.

The distinctive zwitterionic nature of OPDEA, arising from the N^+^-O^−^ structure, imparts a near-neutral charge to the polymer [7]. This property is pivotal in minimizing nonspecific biomolecule adsorption, a common drawback of traditional polymers like polyethylene glycol (PEG). Unlike PEG, which often suffers from rapid clearance by the immune system and limited cell membrane interaction, OPDEA can weakly bind to cell membrane phospholipids via dynamic hydrogen bonds [8]. This interaction triggers transcytosis, an active cellular uptake mechanism that overcomes the limitations of passive diffusion in conventional nanomedicines [9].

In cancer therapy, OPDEA-based nanosystems have demonstrated remarkable potential. They can achieve enhanced tumor penetration through adsorption-mediated transcytosis (AMT) [10]. Moreover, the hypoxia-responsive N-oxides in these nanosystems can be reduced by CYP450 enzymes to protonated tertiary amines, thereby enhancing tumor retention and drug release [11]. In oral drug delivery, OPDEA-PCL micelles have shown advantages by utilizing antimucosal adhesion and nonlysosomal transcytosis pathways [12]. These micelles enable paclitaxel oral formulations to achieve antitumor efficacy comparable to, or even surpassing, that of intravenously administered PEGylated systems [13]. Beyond cancer therapy, these polymers have exhibited exceptional performance in various biomedical applications. In antibiofouling applications, PAO coatings can reduce protein adsorption by up to 90%. In antimicrobial treatments, they can target peptidoglycan to penetrate biofilms. For cardiovascular disease therapy, ROS-sensitive prodrugs based on these polymers show promise in plaque clearance.

Despite these promising advancements, challenges remain in the development of TAO-containing zwitterionic polymers. These include their insufficient biodegradability, potential long-term metabolic toxicity, and issues related to scalability in production. Addressing these challenges is crucial for the translation of these polymers from preclinical research to clinical applications [14].

The purpose of this review is to provide a comprehensive and balanced overview of the current state of the research on TAO-containing zwitterionic polymers. We aim to highlight their unique properties, potential applications, and the mechanisms that underpin their performance. By critically analyzing the existing literature and identifying gaps in the current research, we hope to provide a roadmap for future developments in this exciting field. Through this review, we aim to stimulate further research and drive the development of next-generation nanomedicines that can offer more efficient and precise therapies for a range of diseases.

## 2. Synthetic Strategies and Structural Characteristics

### 2.1. Controlled Polymerization Techniques for TAO-Based Polymer Backbones

The synthesis of tertiary amine oxide-containing zwitterionic polymers like OPDEA involves a combination of controlled polymerization techniques and post-polymerization modifications. Common polymerization methods, including Atom Transfer Radical Polymerization (ATRP), Reversible Addition-Fragmentation Chain Transfer (RAFT) polymerization, and Reversible Deactivation Radical Polymerization (RDRP), are employed to synthesize the precursor polymers with tertiary amine groups. ATRP is a widely used controlled polymerization method that leverages the synergistic action of transition metal catalysts (e.g., copper salts) and ligands to enable controlled monomer polymerization [15]. RAFT polymerization controls the polymerization process through the reversible cleavage and recombination of chain transfer agents [16]. In the synthesis of tertiary amine oxide-based polymers, a suitable RAFT agent is used to initiate the polymerization of monomers containing tertiary amine groups, yielding a precursor polymer. RDRP is a highly efficient and controllable radical polymerization method. By synthesizing a precursor polymer containing tertiary amine groups via RDRP and subsequently introducing N-oxide groups through oxidation, tertiary amine oxide-based polymers with specific structures and properties can be prepared [17]. These methods are chosen because of their ability to provide precise control over the molecular weight and architecture of the polymers, which is crucial for the subsequent introduction of functional groups.

In addition to these methods, other synthetic strategies such as ring-opening polymerization (ROP), post-polymerization modification (PPM), electrochemical polymerization, enzyme-catalyzed polymerization, and click chemistry have also been explored. ROP has been used to synthesize polypeptoids with zwitterionic side chains by initiating the polymerization of N-substituted N-carboxyanhydrides with nucleophilic initiators like primary amines. PPM offers a versatile approach to introduce zwitterionic functionalities after the initial polymerization. For example, poly(N-acryloxysuccinimide) can be modified post-synthesis to introduce zwitterionic groups, allowing for the production of methylated and non-methylated polymers. Electrochemical polymerization and enzyme-catalyzed polymerization provide alternative routes for synthesizing zwitterionic polymers under milder conditions.

After the synthesis of the precursor polymer, the tertiary amine groups are oxidized to form N-oxide groups, which could be reversible under hypoxia conditions in vivo [18]. This oxidation step is pivotal in conferring the zwitterionic nature to the polymer. The choice of oxidizing agent and reaction conditions is carefully optimized to ensure high conversion while maintaining the integrity of the polymer backbone. For example, OPDEA is synthesized by first preparing a tertiary amine-containing precursor polymer via ATRP and then performing oxidative modification [19].

Click chemistry, such as the copper-catalyzed azide–alkyne cycloaddition (CuAAC) reaction, has been employed to functionalize zwitterionic polymers with various ends like tumor-targeting peptides, offering high efficiency and specificity in the modification process.

### 2.2. Polymer Functionalization Design

To further enhance the functionality of OPDEA, block copolymer architectures can be designed. By copolymerizing OPDEA monomers with hydrophobic monomers such as polycaprolactone (PCL), amphiphilic block copolymers are formed. These copolymers can self-assemble into micelles in solution, where the hydrophobic segments form the core and the OPDEA segments constitute the shell [20]. This design not only improves the solubility of hydrophobic drugs but also endows the micelles with the unique properties of OPDEA, such as antifouling effects and cell membrane affinity.

Additionally, stimuli-responsive groups can be incorporated into OPDEA polymers to enable intelligent drug release. For instance, disulfide bonds can be introduced to allow the polymer to cleave in reductive environments, such as the high glutathione environments found in tumor tissues [21]. Similarly, reactive oxygen species (ROS)-sensitive linkages can be included to enable the polymer to degrade in environments with high ROS levels, thereby releasing the drug in a controlled manner. These functionalization strategies significantly expand the versatility of OPDEA in various biomedical applications [22].

## 3. Structural Characteristics

The structural characteristics of OPDEA polymers play a pivotal role in determining their performance in biomedical applications [23]. The zwitterionic nature of OPDEA, arising from the N^+^-O^−^ structure, imparts a near-neutral charge to the polymer. This unique feature minimizes nonspecific biomolecule adsorption, a critical advantage over traditional polymers like PEG. Extensive research has demonstrated that this property enables OPDEA to avoid immune system recognition and clearance, thereby prolonging its circulation time in the bloodstream [24]. For example, studies have shown that OPDEA-modified nanoparticles exhibit significantly reduced protein adsorption compared to PEG-coated nanoparticles, with fibrinogen adsorption levels as low as 5 ng/cm^2^ [25].

The dynamic cell membrane affinity of OPDEA is another key structural characteristic. The N^+^-O^−^ groups in OPDEA can form reversible hydrogen bonds with the phosphocholine headgroups of cell membrane phospholipids. This weak interaction allows OPDEA-based nanocarriers to transiently bind to cell membranes without causing disruption [26]. This property facilitates transcytosis, an active cellular uptake mechanism that enables efficient drug delivery across biological barriers [27]. This interaction strength is sufficient to withstand physiological thermal fluctuations but is reversible under microenvironmental changes, such as a reduction in pH or change in bacterial surface charge.

The structural diversity of OPDEA polymers can be tailored to meet specific application requirements. By varying the monomers used, adjusting the degree of polymerization, or incorporating different functional groups, a wide range of OPDEA variants can be synthesized [28]. This diversity allows for the precise tuning of properties such as the size and stability of micelles formed by block copolymers, as well as the stimuli-responsiveness of the polymers [29]. This flexibility in structural design is crucial for optimizing the performance of OPDEA polymers in different biomedical applications.

### 3.1. Zwitterionic Properties

Tertiary amine oxide-based polymers exhibit unique zwitterionic characteristics due to their N^+^-O^−^ structure, which imparts an overall near-neutral charge. This property minimizes nonspecific biomolecule adsorption, enabling excellent antifouling performance in vivo [30]. By avoiding immune system recognition and clearance, these polymers can circulate in the bloodstream for extended periods. Studies have shown that this near-neutral charge results from the balanced distribution of positive and negative charges within the polymer structure, creating a surface that is less likely to interact with charged biomolecules in the bloodstream [31]. For instance, in physiological environments, the zeta potential of these polymers is close to 0 mV, which significantly reduces the electrostatic interactions with negatively charged serum proteins such as albumin and fibrinogen. This minimizes protein adsorption and prevents the formation of an immune-recognizable surface, thereby prolonging the circulation time of polymer-based nanoparticles in the blood. Furthermore, the hydration layer formed by the zwitterionic groups contributes to steric hindrance and solvation repulsion, which further inhibits biomolecule adsorption. This combination of electrostatic neutrality and hydration layer effects provide a robust mechanism for the antifouling properties of tertiary amine oxide-based polymers, making them highly effective in reducing nonspecific interactions in biological environments [32].

Active transcytosis is a key mechanism through which nanomedicines can overcome tumor barriers. OPDEA polymers enhance tumor penetration via adsorption-mediated transcytosis, utilizing endothelial/epithelial cell transport pathways [33]. Smaller nanoparticles (~30 nm OPDEA-PS micelles) showed threefold higher tumor penetration than 140 nm micelles, due to their ease of uptake via macropinocytosis and rapid transport through cellular organelles [34]. OPDEA’s zwitterionic nature maintains stealth at neutral pH but undergoes charge reversal in acidic tumor microenvironments, further promoting cellular uptake and transport. This size optimization and microenvironment responsiveness offer new strategies for efficient tumor penetration [35].

### 3.2. Dynamic Cell Membrane Affinity

These polymers can weakly bind to cell membrane phospholipids via dynamic hydrogen bonds [36]. This interaction does not disrupt the cell membrane but facilitates intercellular transfer, triggering transcytosis. Transcytosis is an active cellular uptake mechanism that allows the polymer carrier to be rapidly internalized by tumor vascular endothelial cells and tumor cells, and then transported between cells [37]. This overcomes the limitations of passive diffusion in conventional nanomedicines, enabling efficient drug penetration and delivery within tumor tissues. The dynamic hydrogen bonds formed between the zwitterionic groups of the polymer and the phospholipids of the cell membrane are key to this interaction. These bonds are strong enough to initiate the transcytosis process but weak enough to allow the polymer to be released once inside the cell. This delicate balance ensures that the polymers can effectively deliver their payload without causing significant disruption to the cell membrane. The ability to trigger transcytosis is particularly advantageous in tumor therapy, as it allows for deeper tumor penetration and more efficient drug delivery. This mechanism not only enhances the bioavailability of the drug but also reduces the dosage required, potentially minimizing side effects [38].

### 3.3. Topological Variability

Tertiary amine oxide-based polymers exhibit topological variability, which can be tailored by selecting different monomers, adjusting the degree of polymerization, or incorporating various functional groups. This structural diversity enables their broad application in biomedicine, allowing for the design of polymers with specific properties for different applications. For example, modifying the length and type of hydrophobic segments can adjust the size and stability of block copolymer micelles, while introducing different stimuli-responsive groups can enable precise control over drug-release behavior [39].

## 4. Core Structural Advantages and Applications

### 4.1. Zwitterionic Properties to Minimize Nonspecific Protein Adsorption

The zwitterionic nature of tertiary amine oxide-based polymers, imparted by N^+^-O^−^ groups, results in a near-neutral surface charge that minimizes nonspecific protein adsorption. For instance, OPDEA’s N^+^-O^−^ groups maintain a near-neutral charge (ζ-potential close to 0 mV) in physiological environments, weakening electrostatic interactions with negatively charged serum proteins (e.g., albumin, fibrinogen) or mucosal glycoproteins. Dynamic light scattering (DLS) and transmission electron microscopy (TEM) analyses have confirmed that OPDEA-modified nanoparticles remain stable at physiological pH (7.4), with a fibrinogen adsorption rate below 5 ng/cm^2^, outperforming traditional PEG-coated systems [40].

**Figure 1:**
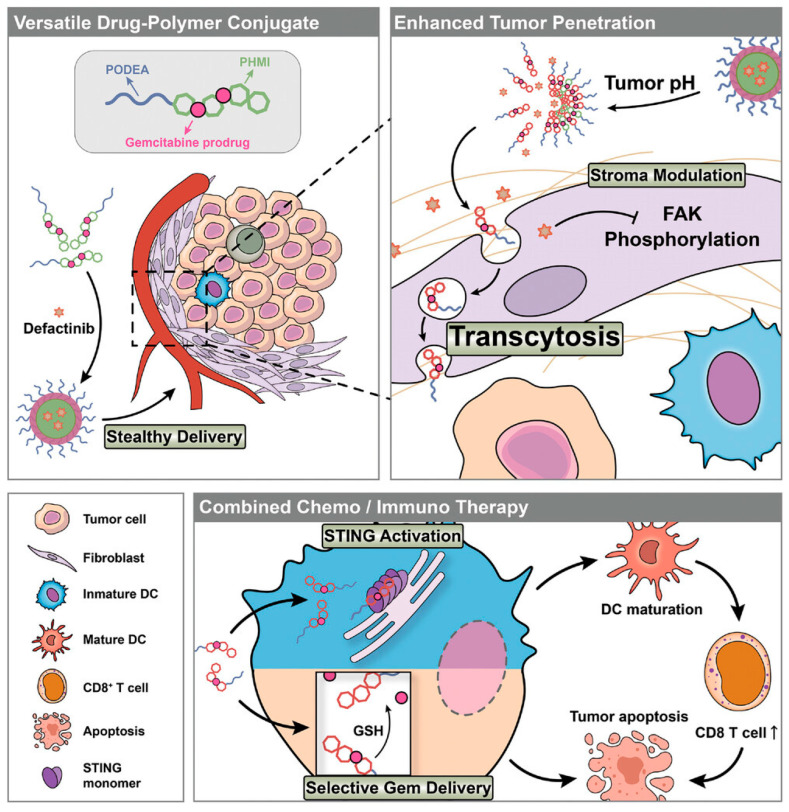
Schematic illustration of transcytosis-mediated tumor penetration, microenvironment acidity, and intracellular GSH dual-responsive drug release profile of PODEA-Gem-HMI nanoparticles, which have stroma modulation and chemo/immunotherapy promotion capacities. Reprinted with permission from Ref. [41]. Copyright © 2023, Wiley.

The molecular mechanism underlying this antifouling property involves the rigid hydration layer formed by N^+^-O^−^ groups (Figure 1), which immobilize 8~12 water molecules via hydrogen bonds, creating a water molecule network with a dissociation energy of up to 15.3 kJ/mol, which is higher than PEG’s 9.2 kJ/mol. This layer hinders protein adsorption through steric hindrance and solvation repulsion [41].

This characteristic extends OPDEA’s applicability to complex biological environments. In oral delivery, OPDEA-PCL micelles reduce adsorption to intestinal mucin (binding constant (K_a_) of 4.77 × 10^3^ M^−1^, half of the K_a_ of PEG-PCL micelles) while maintaining rapid diffusion in mucus, achieving a transmucosal penetration rate of 4.88 μg h^−1^ cm^−2^, which is 2.8-fold higher than that of PEG systems [42]. This dual “antifouling–penetrating” property enhances circulation stability: OPDEA nanoparticles exhibit a 1.5-fold longer plasma half-life than PEG, and OPDEA micelles reside in the intestine for 6 h with 4-fold higher liver enrichment efficiency than PEG. Notably, OPDEA’s weak phospholipid affinity triggers dynamic cell membrane adsorption, promoting trans-epithelial transcytosis and avoiding lysosomal degradation. This “selective antifouling” design overcomes the traditional trade-off between antifouling properties and cellular uptake, offering a new paradigm for nanomedicines in tumor penetration, mucus barrier crossing, and oral delivery.

### 4.2. Dynamic Phospholipid Affinity

TAOs form reversible hydrogen bonds with the phosphocholine headgroups of cell membrane phospholipids. This weak interaction allows nanocarriers to transiently bind to biomembranes while maintaining flexibility to dissociate in specific microenvironments. For example, OPDEA’s tertiary amine oxide groups form hydrogen bonds with phosphate groups on phospholipid heads, with bond strengths of 10~30 kJ/mol, which is sufficient to withstand physiological thermal fluctuations but is reversible under microenvironmental changes (e.g., reduction in pH or change in bacterial surface charge) [43].

This dynamic binding enables nanocarriers to stably adsorb onto red blood cell surfaces during circulation while rapidly responding at lesion sites. It is crucial to prioritize specific toxicity mechanisms like hepatic accumulation and immune activation to reduce the risk upon clinical translation. Compared to PEG or poly(sulfobetaine), TAO polymers exhibit lower hepatic accumulation and reduced immune activation. For instance, OPDEA micelles leverage erythrocyte hitchhiking to prolong circulation, achieve targeted release via specific lesion binding, and enhance biomembrane penetration through charge conversion in acidic microenvironments [44]. This multidimensional mechanism improves drug delivery efficiency and reduces off-target tissue toxicity.

RBC-hitchhiking is an innovative strategy where nanoparticles adsorb onto the RBC surface, significantly prolonging their circulation time. The negative charge of RBCs interacts electrostatically with nanoparticles, enabling this mechanism. TAO-modified liposomes (TAOLs) leverage RBC-hitchhiking to achieve extended circulation and detachment at tumor vasculature to enter tumor tissues. This avoids rapid nanoparticle clearance and enhances tumor penetration. In mouse models, TAOLs demonstrated excellent circulation stability, with pharmacokinetic parameters (e.g., AUC and t_1/2_) comparable to traditional liposomes but with significantly improved tumor accumulation. The simplicity and universality of this strategy make it highly effective for extended circulation and tumor targeting [45].

Mitochondria-targeted therapy delivers drugs precisely to tumor cell mitochondria [46]. For example, a polymer–drug conjugate (OPDMA-Cela) links celastrol to a zwitterionic polymer, improving its water solubility and circulation time [47]. This conjugate disrupted the mitochondrial membrane potential, inducing immunogenic cell death (ICD) [48] and downregulating PD-L1. This approach addresses celastrol’s bioavailability challenges and provides a versatile platform for mitochondrial-targeted anticancer drugs [49].

Atherosclerosis is a chronic inflammatory cardiovascular disease characterized by lipid and immune cell deposition [50]. A nano-prodrug (OPDH-SV) links simvastatin to a tertiary amine oxide zwitterionic polymer via ROS-responsive oxalate ester bonds. In vitro, OPDH-SV exhibited stability, low toxicity, and long circulation, reducing the levels of intracellular ROS. In vivo, it significantly reduced the plaque area and ROS levels in ApoE−/− mice, demonstrating therapeutic potential with good safety [51]. The enhanced therapeutic efficacy is primarily attributed to the extended circulation time provided by the TAO polymer, which allows for prolonged exposure of the drug to the atherosclerotic lesions. While the nano-prodrug shows potential for enhanced atherosclerosis therapy, the evidence for specific targeting is limited. The improved outcomes are more accurately attributed to the polymer’s ability to prolong drug circulation and facilitate interaction with the disease microenvironment rather than specific targeting mechanisms.

TAO zwitterionic polymers enhance oral drug absorption by interacting with gastrointestinal mucus and epithelial cells. They form stable adsorption layers with mucus, overcoming mucosal barriers, and bind specifically to epithelial cell membranes, promoting transmembrane transport [52]. This improves bioavailability, reduces gastrointestinal degradation, and ensures drug release under gastric acid and enzymatic conditions. These polymers show great potential for oral drug delivery in cancer and chronic disease management [53]. Prioritizing toxicity mechanisms like hepatic accumulation and immune activation is essential for clinical translation. OPDEA polymers compare favorably to PEG or poly(sulfobetaine) in these aspects, showing lower hepatic accumulation and reduced immune activation.

Tertiary amine oxide zwitterionic polymers improve ocular drug delivery by interacting with corneal and conjunctival tissues [54]. They bind to corneal epithelial cell membranes, extending drug retention and enabling corneal penetration. This enhances bioavailability, reduces drug degradation, and ensures sustained release in ocular environments [55]. These polymers show significant potential for treating glaucoma and dry eye disease [56].

### 4.3. Microenvironment Responsiveness

Under hypoxic conditions, N-oxide groups can be bio-reduced to tertiary amines by CYP450 enzymes [57], and further protonated to positively charged quaternary ammonium groups in the acidic tumor microenvironment, enhancing cellular adsorption.

In hypoxic tumor regions, overexpressed CYP450 enzymes catalyze the reduction of N-oxide bonds to generate tertiary amine structures. Under hypoxic conditions with CYP450 and NADPH, the characteristic NMR signals of N-oxides (3.3 ppm and 3.7 ppm) disappear, while tertiary amine signals (2.9 ppm and 3.5 ppm) emerge [58]. ζ-potential measurements confirmed charge reversal from −15 mV to +8 mV. This reduction is tumor-specific, as oxygen-rich normal tissues suppress CYP450 activity, minimizing off-target effects.

The generated tertiary amines protonate in the acidic tumor microenvironment (pH 6.2–7.1), forming positively charged quaternary ammonium groups [59]. In our previous study, OPDEA nanoparticles exhibited a ζ-potential shift from −3 mV to +18 mV at pH 6.8, promoting electrostatic binding to negatively charged tumor cell membranes (e.g., phospholipid heads or sialic acid residues) and adsorption-mediated transcytosis [60]. This dynamic charge property enables nanoparticles to penetrate dense tumor matrices, achieving 4.3 times deeper penetration in melanoma multicellular spheroids than PEG systems. Inhibition of CYP450 significantly reduces penetration efficiency, underscoring the necessity of enzymatic responsiveness [61].

**Figure 2:**
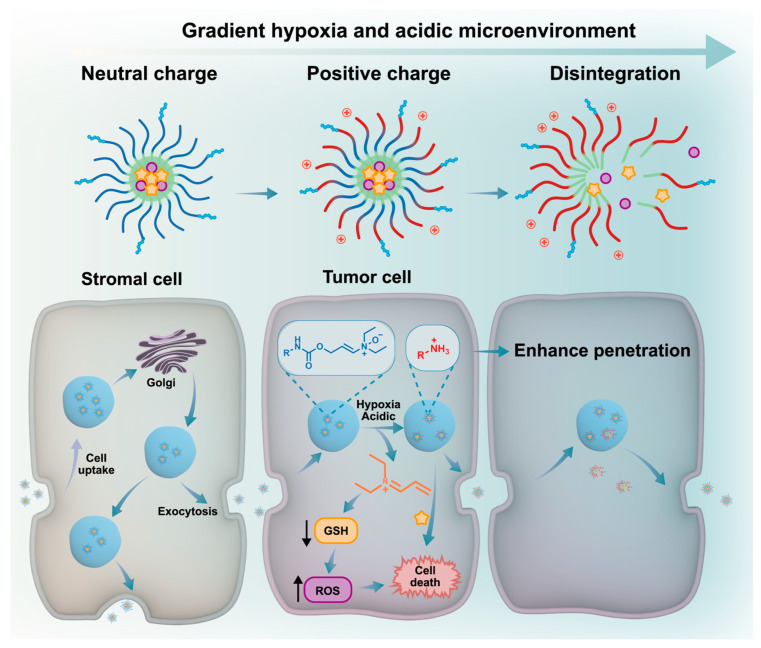
Schematic illustration of transcytosis-triggering nanoparticles for overcoming stromal barriers, responding to hypoxia microenvironment, and reversing immunosuppression in pancreatic cancer combinatorial therapy [35]. Reprinted with permission from Ref. [35]. Copyright © 2025, American Chemical Society.

N-oxides are reduced to tertiary amines in hypoxic tumor regions, enhancing drug retention and enabling imaging. In pancreatic ductal adenocarcinoma (PDAC), dense stromal barriers hinder drug penetration and immune cell infiltration while paradoxically restraining tumor spread [62]. This study developed hypoxia/acid-responsive nanoparticles co-loaded with a gemcitabine prodrug and TGF-β/SMAD inhibitor galunisertib, utilizing amphiphilic amino acid polymers modified with enamine N-oxides (Figure 2). The nanoparticles exploit transcytosis via phospholipid interactions and undergo charge reversal in tumor microenvironments to enhance deep tissue penetration. Galunisertib suppresses stromal activation and immune suppression by blocking pSMAD2/3 signaling, synergizing with released gemcitabine to eradicate cancer cells. This dual-action strategy simultaneously overcomes physical/biological stromal resistance while enabling targeted chemotherapy and immune modulation.

In another work, the OPDEA/BOD-NO_2_ probe achieved 48 h dual-channel hypoxia ratio imaging. In hypoxic environments, its fluorescence shifts from strong red light (λex = 660 nm, λem = 710 nm) to strong NIR fluorescence (λex = 720 nm, λem = 790 nm), providing two imaging channels. Compared to PEG/BOD-NO_2_, OPDEA/BOD-NO_2_ showed improved tumor accumulation and signal-to-background ratios, with high water solubility, specificity, and biocompatibility, showing promise for clinical hypoxia tumor diagnosis and therapy monitoring [63].

## 5. Challenges and Future Outlook

Most tertiary amine oxide-based polymers lack hydrolytically labile groups, leading to slow in vivo degradation. While the slow biodegradability of most TAO-based polymers raises concerns about potential long-term physiological toxicity due to polymer accumulation within the body, this is particularly relevant for applications like antimicrobial coatings and oral nanomedicines where polymers may be excreted or accumulate in specific tissues. Future designs could incorporate biodegradable backbones (e.g., PEG-PLA, PBAE) with dynamic covalent bonds (e.g., disulfides, Schiff bases) for responsive disassembly [64]. Degradation products like CO_2_ and H_2_O could be achieved using poly(N-oxide-caprolactone) (PNO-PCL) triggered by esterases [65]. Degradable backbones can reduce long-term toxicity concerns by allowing the polymers to break down into less harmful products after fulfilling their drug delivery function. For instance, incorporating a degradable backbone into OPDEA could enable the polymer to facilitate drug delivery to a tumor site and then be metabolized and excreted, minimizing the risk of accumulation in the body. Such biodegradable designs can help balance therapeutic efficacy with safety by allowing the polymers to break down into less harmful products after fulfilling their function, thereby minimizing the risk of physiological toxicity from polymer accumulation.

High-molecular-weight polymers can accumulate in unexpected organs, causing unclear long-term toxicity [66]. Long-term animal models combined with mass spectrometry imaging (MSI) and radiolabeling (e.g., ^14^C) are needed to track metabolic pathways and degradation products. Organ-on-a-Chip models could assess metabolic impacts on organ function [67]. Organ-specific modules can enhance targeting accuracy by directing the nanomedicine to the desired site of action with greater precision.

The current synthesis routes are multi-step and low-yield. One-pot controlled polymerization like RAFT with in situ oxidation or microfluidic continuous production could enhance batch consistency and drug loading [68]. Microreactor technology could streamline ATRP and oxidation steps and meet FDA GMP standards [69]. Intelligent production methods could improve the consistency and scalability of manufacturing, ensuring that these advanced materials can be reliably produced for clinical use.

## 6. Conclusions

TAO-based polymers represent a significant advancement in nanomedicine delivery through their dynamic interfacial interactions and microenvironment responsiveness. Throughout this review, we highlighted their distinctive properties, such as their zwitterionic nature that minimizes nonspecific protein adsorption and their dynamic cell membrane affinity that facilitates active transcytosis. These characteristics have been shown to enhance drug delivery efficiency in various biomedical applications, including tumor penetration and oral bioavailability. These polymers offer promising alternatives to traditional materials like PEG, particularly in specialized applications where their unique properties can be leveraged.

While challenges in degradability, toxicity, and manufacturing persist, the integration of these elements could enhance the precision and safety of nanomedicine delivery systems. This approach has the potential to move nanomedicine closer to active delivery systems, where the nanomedicine actively responds to the biological environment and delivers the drug in a controlled and targeted manner. We remain optimistic about the future developments in this field and the potential for OPDEA to contribute to more effective and safer therapeutic options, particularly for solid tumor therapy.

## Data Availability

Not applicable.
